# Ponatinib vs. asciminib in post–second-generation tyrosine kinase inhibitor therapy for chronic-phase chronic myeloid leukemia: a matching-adjusted indirect comparison

**DOI:** 10.3389/fonc.2024.1455378

**Published:** 2024-11-20

**Authors:** Valentin Garcia-Gutierrez, Fei Huang, Ajibade Ashaye, Mehul Dalal, Victor Laliman-Khara, Massimo Breccia, Megan Rutherford, Hoora Moradian, Petros Patos, Elias Joseph Jabbour

**Affiliations:** ^1^ Ramón y Cajal University Hospital, Instituto Ramón y Cajal de Investigación Sanitaria (IRYCIS), Universidad de Alcalá, Madrid, Spain; ^2^ Global Evidence and Outcomes Oncology, Takeda Development Center Americas, Inc, Lexington, MA, United States; ^3^ Advanced Analytics Advisory, HEOR, Cytel Inc., Waltham, MA, United States; ^4^ Department of Translational and Precision Medicine, Sapienza University, Rome, Italy; ^5^ Comparative Effectiveness, Cytel Inc., Waltham, MA, United States; ^6^ Hematology, Region Europe, Incyte Biosciences International Sàrl, Morges, Switzerland; ^7^ Department of Leukemia, Division of Cancer Medicine, The University of Texas MD Anderson Cancer Center, Houston, TX, United States

**Keywords:** chronic myeloid leukemia, ponatinib, asciminib, T315I mutation, BCR::ABL1 (BCR-ABL1), major molecular response

## Abstract

**Background:**

Ponatinib and asciminib are approved for third-line therapy in chronic-phase chronic myeloid leukemia (CP-CML) and are the only drugs approved for patients with the T315I mutation in the United States. In Europe, only ponatinib is approved for patients with the T315I mutation.

**Methods:**

Clinical trials evaluating ponatinib or asciminib in patients with relapsed and refractory (R/R) CP-CML who failed one or more second-generation TKIs or had the T315I mutation were identified in a systematic review of medical literature databases. A matching-adjusted indirect comparison (MAIC) analysis with individual patient-level data with ponatinib was used to balance baseline characteristics between ponatinib and asciminib groups. After matching, the response rate was calculated using the MAIC weight for each patient and the difference in response rate was calculated using a two-independent proportion Z-test. Cumulative rates of *BCR::ABL1* ^IS^ ≤1% and major molecular response (MMR) in patients without baseline response were compared. Patients were further stratified by T315I mutation status.

**Results:**

The MAIC included four trials (ponatinib: NCT02467270, NCT01207440; asciminib: NCT02081378, NCT03106779). In patients without baseline response of *BCR::ABL1* ^IS^ ≤1%, the adjusted *BCR::ABL1* ^IS^ ≤1% rate difference with ponatinib vs. asciminib was 9.33% (95% confidence interval [CI]: 0.79%–17.86%; adjusted MMR rate difference: 6.84% [95% CI: −0.95%–14.62%]) by 12 months in favor of ponatinib. In patients with the T315I mutation, adjusted *BCR::ABL1* ^IS^ ≤1% rate difference with ponatinib vs. asciminib was 43.54% (95% CI: 22.20%–64.87%; adjusted MMR rate difference: 47.37% [95% CI: 28.72%–66.02%]) by 12 months.

**Conclusion:**

After key baseline characteristics adjustment, cumulative *BCR::ABL1* ^IS^ ≤1% and MMR rates were statistically higher with ponatinib than asciminib in patients without a baseline response in most of the comparisons by 12 months. Favorable efficacy outcomes observed in ponatinib vs. asciminib were consistently stronger in the T315I mutation subgroup.

## Introduction

Chronic myeloid leukemia (CML) is a type of myeloproliferative neoplasm with an incidence of between 1 and 2 per 100,000 persons per year (1). An estimated 1310 Americans died of CML in 2023 (1). CML is classified into three phases: chronic phase (CP-CML), accelerated phase (AP-CML), and blast phase (BP-CML). CP-CML is the least advanced phase of CML, where blasts in bone marrow or blood samples were <10%. In AP-CML, patients have between 15% and 30% blasts in their samples, and basophils comprise 20% of the blood. In BP-CML, patients have ≥30% blasts, and large clusters are observed in the bone marrow (2). Based on data up to 2018, the 5-year survival rate for CML is 70% (1). However, phase-specific survival statistics are lacking.

The National Comprehensive Cancer Network® (NCCN®) recommends targeted treatment with a tyrosine kinase inhibitor (TKI) as an option in first-line treatment of CML (3).^1^ Since the introduction of TKIs to treat CML, the 5-year survival rate has improved by over three times since the mid-1970s, when the survival rate was 22% (1). In an analysis of 483 patients with newly diagnosed CP-CML enrolled in six parallel prospective trials from 2000 to 2012, the 5-year relative survival rate was 95% (4).

TKIs for CML work by binding to the adenosine triphosphate (ATP) site of the BCR::ABL1 oncoprotein to inhibit aberrant kinase activity (5, 6). Imatinib (7) was the first TKI used for the treatment of CML, followed by the second-generation (2G) TKIs nilotinib (8, 9), bosutinib (10), and dasatinib (11). Although initial response rates are high with imatinib, up to 40% of patients fail (12). These patients may respond to treatment with 2G TKIs; however, 37%–52% of patients do not have a response (10, 11, 13). The third-generation (3G) TKI ponatinib inhibits the unmutated and mutated *BCR::ABL* gene, including the threonine-to-isoleucine mutation at position 315 (T315I) (14), which is present in nearly one third of patients with resistant disease (15, 16). In a long-term, 5-year follow-up study, ponatinib was shown to be effective in patients who had received prior therapies and was associated with favorable long-term survival outcomes (17). In addition, asciminib is a novel ABL myristoyl pocket (STAMP) inhibitor that restores the negative regulator functions of *ABL1* (5, 6, 18) and maintains activity against most *ABL1* mutations, such as T315I (18–21).

While the evolution of TKIs has improved the survival of patients with CP-CML, resistance and intolerance are persistent challenges. Ponatinib and asciminib are both indicated for third-line therapy and are the only TKIs indicated for the T315I mutation in CP-CML in the United States. Both ponatinib and asciminib have demonstrated efficacy in major molecular response (MMR) and/or complete cytogenetic response (CCyR) in resistant and intolerant CP-CML.

In a phase 2 trial of ponatinib (PACE), patients with CML with resistance to or intolerance of dasatinib or nilotinib or with the T315I mutation were administered 45 mg ponatinib daily. Among those with CP-CML, 46% had a complete cytogenetic response by 12 months (22). In another phase 2 trial of ponatinib (OPTIC), which introduced response-based dosing, patients with CP-CML resistant to or intolerant of ≥2 prior BCR::ABL1 TKIs or with a *BCR::ABL1* T315I mutation were randomized to receive three different starting doses (45, 30, or 15 mg once daily) of ponatinib and in the 45-mg and 30-mg cohorts, doses were reduced to 15 mg upon achievement of *BCR::ABL1* transcript on the international scale (*BCR::ABL1* ^IS^) ≤1%. *BCR::ABL1* ^IS^ was used to determine primary and secondary endpoints. The primary endpoint of *BCR::ABL1* ^IS^ ≤1% at 12 months was achieved in 44.1% (45-mg cohort), 29.0% (30-mg cohort), and 23.1% (15-mg cohort) of patients (16). In a phase 1, dose-escalation study of asciminib, patients with CML with resistance or intolerance to ≥2 ATP-competitive TKIs were administered asciminib at doses of 10–200 mg once or twice daily. In the evaluable patients with CP-CML, regardless of their *BCR::ABL1* baseline level, 48% achieved or maintained MMR by 12 months. For the patients with CP-CML with the T315I mutation, MMR was achieved or maintained in five patients (28%) by 12 months regardless of their baseline level of *BCR::ABL1* ^IS^, although a five-fold dose was required to achieve similar efficacy as in patients without the T315I mutation (18). Additionally, in a phase 3 trial of asciminib (ASCEMBL), patients with CP-CML previously treated with ≥2 TKIs were randomized to receive 40 mg asciminib twice daily or 500 mg bosutinib once daily. The MMR rate at Week 24 was 25.5% with asciminib and 13.2% with bosutinib (20).

The efficacy of ponatinib treatment for patients with CP-CML has not been compared with asciminib treatments in head-to-head clinical trials. Due to the heterogeneity of patient characteristics in the trials that could potentially influence the efficacy outcomes, we aimed to use the population-matching approach to balance key baseline differences between trials. We evaluated the efficacy of ponatinib compared with asciminib in patients with TKI-resistant CP-CML from various trials using a matching-adjusted indirect comparison (MAIC).

## Materials and methods

### Systematic literature review

A systematic literature review (SLR) was conducted to evaluate the efficacy of 2G and 3G TKIs in patients with CP-CML. Major medical literature databases, including MEDLINE, EMBASE, and the EBM Reviews Collection, were queried using systematic search strategies to identify English language publications from 01 January 2006 to 26 October 2021. Hand searches of select conferences not yet indexed in EMBASE were also conducted. Conference abstracts, including those indexed in databases, were eligible if published in or after the year 2018. Follow-up publications for studies identified in the search of SLR were also included after the SLR cutoff date.

Studies reporting CCyR, MMR, or *BCR::ABL1* ^IS^ ≤1% for patients with CP-CML treated with TKI whose disease was resistant or intolerant to at least one 2G TKI or who had the T315I mutation were included. Updated data from the included trials captured in the SLR were also extracted when available after the SLR cutoff date through hand searches. Only trials including ponatinib and asciminib treatment arms were selected for the MAIC analysis.

### Matching-adjusted indirect comparison

This study employed a MAIC methodology to provide insights into the treatment outcomes for patients with CP-CML. The SLR revealed significant heterogeneity in patient populations across different trials, particularly those in later lines of treatment. Consequently, conducting a conventional network meta-analysis was deemed unfeasible due to the potential introduction of bias resulting from baseline characteristic imbalances. The primary focus of this analysis was to compare trials involving asciminib and ponatinib, two newer CP-CML treatments lacking direct head-to-head trial data. The outcomes of interest for this indirect treatment comparison were *BCR::ABL1* ^IS^ ≤1% and MMR (defined as *BCR::ABL1* ^IS^ ≤0.1%). These outcomes were primary or secondary endpoints in the trials (16–18, 20), providing sufficient maturity and evidence for the application of MAIC. Response data were assessed at the 12-month timeframe to ensure data maturity and align with intended clinical trial endpoints, and in consideration of expert opinion on the optimal evaluation period for CP-CML treatment response. Additionally, a sensitivity analysis was conducted using a 6-month timeframe, as all included studies reported results at this timepoint.

The MAIC analysis followed the method developed by Signorovitch et al. (23) and adhered to the recommendations and framework provided by the National Institute for Health and Care Excellence Decision Support Unit (24). All analyses were conducted using R v4.2.1 and MAIC package v1.4. Initially, clinical experts reviewed baseline characteristics, identifying potential treatment effect or prognostic factors, all of which were considered for adjustment. Key prognostic factors and effect modifiers identified by these experts for population adjustment included age, sex, race, Eastern Cooperative Oncology Group (ECOG) performance status, number of prior TKI treatments, baseline *BCR::ABL1* ^IS^ transcript levels, and resistance or intolerance to prior TKIs. Since there were no common treatment arms between asciminib and ponatinib trials, only an unanchored MAIC was feasible, necessitating the adjustment of both treatment effect modifiers and prognostic factors. The MAIC aimed to adjust imbalances in as many of these factors as possible while maximizing the effective sample size (ESS), which equates to the number of individual (unweighted) patients that would yield the same level of uncertainty in the estimates as the weighted cohorts.

## Results

### Systematic literature search

**Figure 1:**
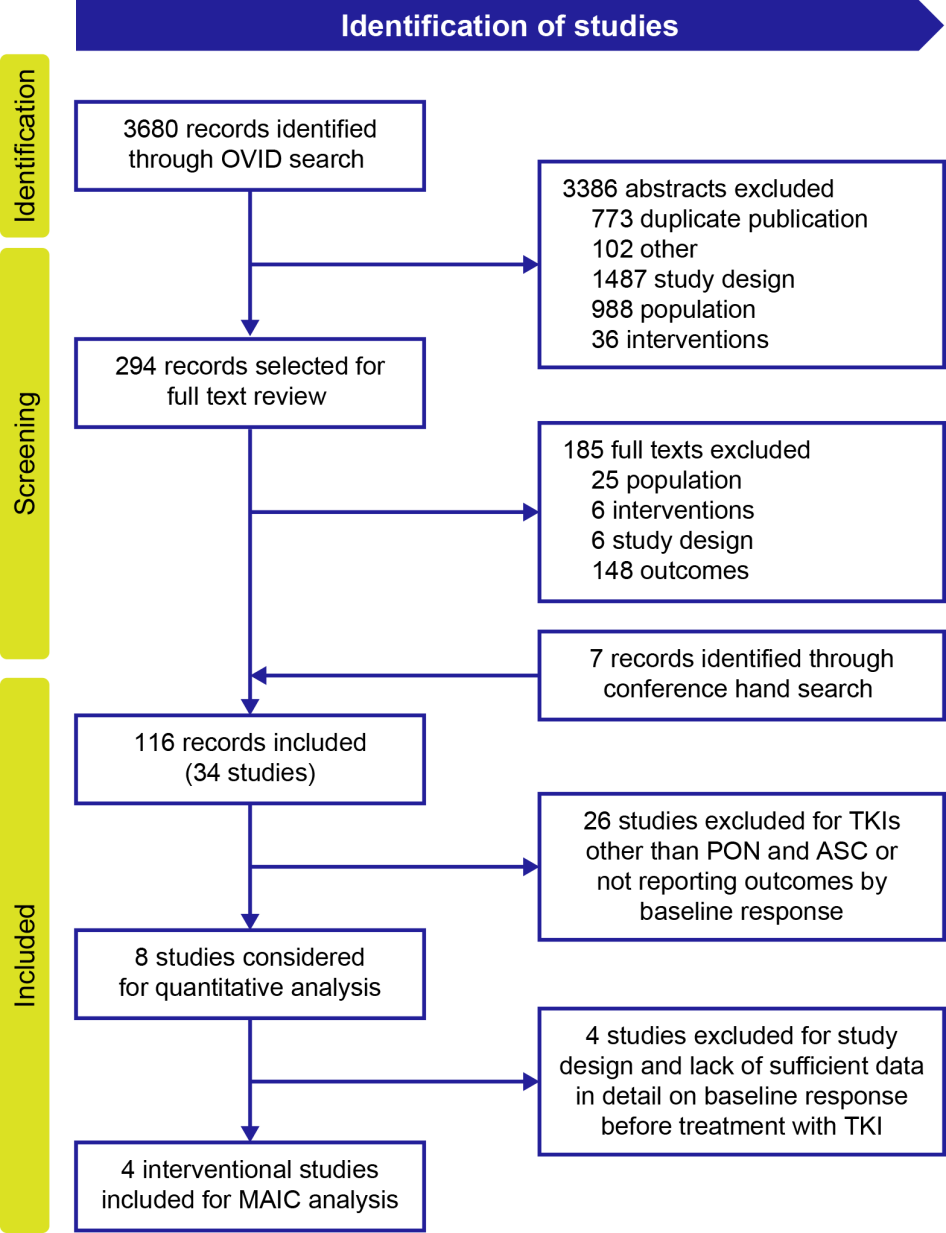
PRISMA flow diagram of included studies for MAIC analysis. ASC, asciminib; MAIC, matching-adjusted indirect comparison; PON, ponatinib; TKI, tyrosine kinase inhibitor.

Of the 3680 publications identified for all TKIs in patients with CP-CML, 116 publications (from databases and conferences) describing 34 unique studies were included in the evidence set for all TKIs. Twenty-six studies were excluded from this evidence set for reporting TKIs other than ponatinib and asciminib or for not reporting outcomes by baseline response (**Figure 1**). Of the remaining eight studies, four (16–18, 20, 22) reported availability of data on response before treatment with a TKI and were selected for the MAIC analysis to compare ponatinib and asciminib among resistant or intolerant patients who did not achieve a baseline response and patients with the T315I mutation in the final evidence set for assessment of *BCR::ABL1* ^IS^ ≤1% and MMR.

A summary of the study and patient characteristics of the included studies is presented in **Table 1**. Trials on asciminib included a phase 1 RCT (NCT02081378) (18) and the phase 3 ASCEMBL randomized clinical trial (RCT; NCT03106779) (20). For ponatinib, the phase 2 PACE (NCT01207440) (22) and OPTIC (NCT02467270) (16) trials were included.

**Table 1:** Summary of study and patient characteristics of the included studies.

Study	Study design	Intervention	N	Age, years, median (range)	Exposure to prior regimens (resistance/ intolerance)	T315I mutation	CCyR at study entry	Study follow-up or treatment duration
**Phase 1 asciminib** (18)	Open-label, phase 1, dose-escalation trial	Asciminib: 10 mg to 200 mg BID and 80 mg to 200 mg QD, orally	141	Non-T3151: 56 (25–88) T315I: 54 (23–76)	Resistance or intolerance to ≥2 prior TKIs	Included (n=28)	Included	Non-T315I: Median follow-up: 72 months (range: 0.1–167) T315I: Median follow-up: 37 months (range: 0.7–167)
**ASCEMBL** (20, 38)	Open-label phase 3, RCT	Asciminib: 40 mg BID, orally	157	52 (24–83)	Resistance or intolerance to ≥2 prior TKIs or intolerance to the previous TKI therapy at time of screening	Excluded	Included	Median follow-up: 27.6 months Median duration of treatment: 23.7 months (range: 0.0–46.3)
**PACE** (22, 39)	Phase 2, single-arm trial	Ponatinib: 45 mg QD, orally	270	58 (18–94)	Resistance or intolerance to dasatinib or nilotinib	Included (n=64)	Excluded	Median follow-up: 56.8 months (range: 0.1–73.1) Median duration of treatment: 32.1 months (range: 0.1–73.0)
**OPTIC** (16, 39)	Open-label, phase 2, single-arm trial	Ponatinib: 45 mg QD and dose reduction to 15 mg QD once achievement of ≤1% *BCR::ABL1*, orally	94	47 (19–81)	Resistance or intolerance to ≥2 prior TKIs	Included (n=25)	Excluded	Median follow-up: 32 months (range: 1–57 months) Median duration of treatment: 19.6 months (range: 0.1–51.3)

BID, twice daily; CcyR, complete cytogenetic response; QD, once daily; RCT, randomized clinical trial; TKI, tyrosine kinase inhibitor.

### Matching-adjusted indirect comparison

The MAIC analysis included a comprehensive examination of individual patient–level data extracted from both the PACE and OPTIC trials. The primary objective was to establish a robust match with the patient characteristics observed in the asciminib phase 1 and ASCEMBL trials. This matching process incorporated key factors such as age, sex, race, ECOG performance status, number of prior TKIs, and the baseline *BCR::ABL1* ^IS^ transcript level before treatment, as presented in **Table 2**. A backward approach was employed until the most influential variables were retained, ensuring model convergence. Variables were chosen based on their impact on achieving MMR and their role in addressing the heterogeneity of treatment effects, particularly concerning the intent-to-treat (ITT) population. Despite adjustment for these factors, variables related to resistance and intolerance to prior TKIs could not be integrated into the MAIC model, due to lack of sufficient intolerant patients in both the PACE and OPTIC trials for ponatinib. The selection of variables for inclusion in the model followed a systematic approach, involving a forest plot and clinical perspective.

**Table 2:** Baseline characteristics of asciminib trials (phase 1 and ASCEMBL) vs. the MAIC-unadjusted and -adjusted ponatinib trials (PACE and OPTIC).

	Phase 1 asciminib	ASCEMBL asciminib	Phase 1 asciminib and ASCEMBL^a^	PACE + OPTIC ponatinib – unadjusted	PACE + OPTIC matching-adjusted^b^
Sample size	141	157	298	359	ESS^c^: 304.97 PACE: 223.32 OPTIC: 81.65
Mean age, years (SD)	55.5^d^	51.0 (13.5)	52.6 (13.5)	55.2 (15.6)	52.6 (13.5)
Sex, male	54.5%	52.2%	53.0%	53.2%	53.0%
Race, White	UNK	75.2%	75.2%	79.9%	75.2%
ECOG performance status, 1 or 2	27.3%	19.1%	22.8%	28.1%	22.8%
Mean prior TKIs (SD)	2.7^e^	2.5 (0.7)	2.6 (0.7)	2.6 (0.7)	2.6 (0.7)
Resistant to prior TKI	NR	60.5%	NA	84.4%	Not adjusted
*BCR::ABL1* ^IS^ level >10%	43.3%	61.8%	55.2%	76.6%	55.2%

ECOG, Eastern Cooperative Oncology Group; ESS, effective sample size; IS, international scale; MAIC, matching-adjusted indirect comparison; NA, not applicable; NR, not reported; SD, standard deviation; TKI, tyrosine kinase inhibitor; UNK, unknown.

a The weighted results from phase 1 and ASCEMBL trial were used as the reference of the MAIC analysis.

b MAIC analysis was conducted by using patient-level data from PACE and OPTIC trial which were matched against the combined results of phase 1 asciminib and ASCEMBL trial in all of the patient characteristics listed in the table.

c Effective sample size: calculated as the square of the summed weights divided by the sum of the squared weights.

d Only median age was available in phase 1 asciminib.

e Prior TKI number in phase 1 asciminib trial was estimated based on the published categorical data.

**Figure 2:**
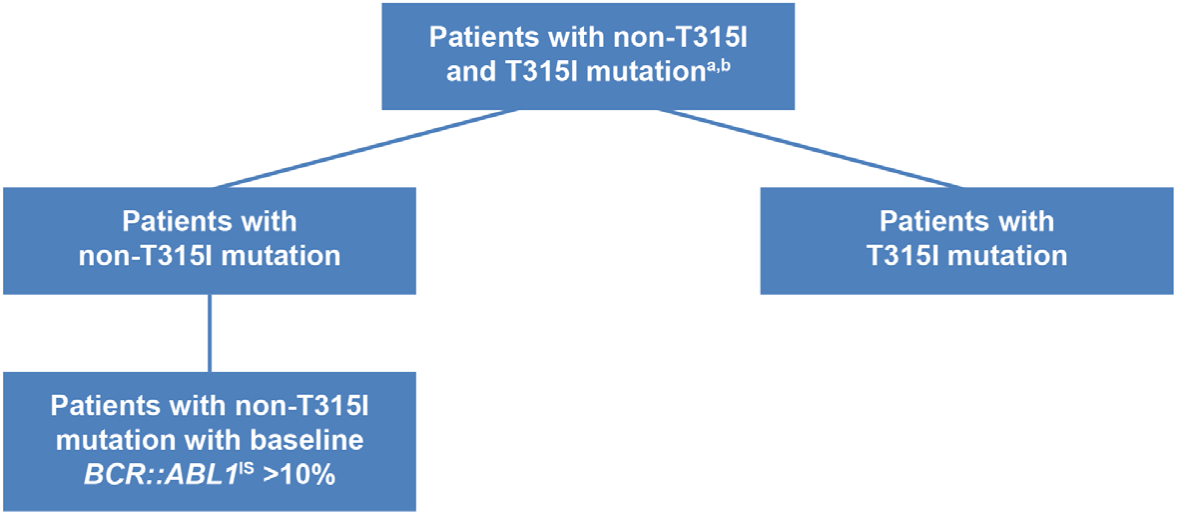
Overall and subgroups of patients with CP-CML with post-2G TKI therapy. ^a^Patients with CP-CML from the ponatinib trials and asciminib trials were matched on age, gender, race, Eastern Cooperative Oncology Group performance status (1, 2, vs. 0 or missing), number of prior TKI, baseline *BCR::ABL1* ^IS^ (>10% vs. ≤10%). Patients with baseline response *BCR::ABL1* ^IS^ ≤1% were excluded in the analysis. ^b^Trials included in the analysis were: asciminib phase 1, open-label, dose-escalation trial (NCT02081378); ASCEMBL phase 3, open-label, randomized clinical trial (NCT03106779); PACE phase 2, open-label trial (NCT01207440); OPTIC phase 2, open-label, dose-optimization trial (NCT02467270). 2G, second-generation; CP-CML, chronic-phase chronic myeloid leukemia; TKI, tyrosine kinase inhibitor.

As both PACE and OPTIC trials did not consider patients who already had *BCR::ABL1* ^IS^ ≤1% prior to treatment as positive responders, our MAIC analysis excluded patients who had achieved a baseline response in all of the selected trials for fair comparisons. Patients identified as having no baseline response in the selected trials were subsequently stratified into subgroups, distinguishing between those with and without the T315I mutation. Another subgroup analysis was conducted for patients exhibiting a higher *BCR::ABL1* ^IS^ level at baseline (*BCR::ABL1* ^IS^ >10%), as illustrated in **Figure 2**. Given that ponatinib and asciminib stand as the sole treatments indicated for CP-CML with the T315I mutation, further subgroup analyses were conducted in patients with and without the T315I mutation.

### Baseline characteristics

Prior to matching on baseline characteristics, compared with asciminib patients, ponatinib patients had higher mean age and were more likely to be White and/or have an ECOG performance status of 1 or 2. Over 20% more ponatinib trial patients had *BCR::ABL1* ^IS^ levels >10% (**Table 2**). A similar proportion of patients in both treatment groups were male, and both groups had similar mean number of prior TKIs. The proportion of patients resistant to prior TKI in the ASCEMBL asciminib trial (60.5%) was substantially lower compared with those in the PACE and OPTIC trials (84.4%). As individual patient-level data were available for the PACE and OPTIC trials only, it was not feasible to match on the percentage of patients who were intolerant in the asciminib trials from those patients who were intolerant in these two trials. After matching adjustment, the ESS of ponatinib patients in PACE and OPTIC decreased from 359 to 305 (**Table 2**).

For patients with CP-CML without a baseline response and the subgroup of patients without the T315I mutation, patients randomized to ponatinib from PACE and OPTIC studies were matched on the prognostic factors from patients of the asciminib trials listed in **Table 2** (exceptions noted in the table). After matching, the covariates used for adjustment in **Table 2** were balanced between the ponatinib and asciminib cohorts.

A subgroup analysis was conducted for patients with the T315I mutation, which was only available in the phase 1 trial for asciminib. Comparison of patient characteristics between phase 1 asciminib and combined PACE and OPTIC (unadjusted and MAIC-adjusted) is presented in **Supplementary Table S1**. The ESS for PACE and OPTIC was reduced from 88 to 53 after MAIC adjustment.

### Comparative efficacy

The original, unadjusted proportions of patients with *BCR::ABL1* ^IS^ ≤1% and MMR in the four included studies are presented in **Table 3** (16, 18, 20, 22). Sample sizes ranged from 87 in the phase 1 asciminib trial to 253 in the PACE ponatinib trial. Both *BCR::ABL1* ^IS^ ≤1% and MMR rates observed by 6 months seemed to be sustained by 12 months. By 12 months, the proportion of patients with *BCR::ABL1* ^IS^ ≤1% ranged from 39.08% in the phase 1 asciminib trial to 52.22% in the OPTIC ponatinib trial. The proportion of patients reaching MMR ranged from 18.89% (95% confidence interval [CI]: 14.14%–23.80%; OPTIC ponatinib trial) to 33.12% (95% CI: 25.36%–40.84%; ASCEMBL asciminib trial) by 12 months. With the exception of MMR by 12 months, the proportion of patients reaching *BCR::ABL1* ^IS^ ≤1% and MMR was lowest in the phase 1 asciminib trial.

**Table 3:** Original *BCR::ABL1* ^IS^ ≤1% and MMR by 6 and 12 months among patients with CP-CML without baseline response.

Intervention	Phase 1	ASCEMBL	PACE	OPTIC
	Asciminib	Asciminib	Ponatinib	Ponatinib
Sample size	N=87	N=142	N=253	N=90
6 months, % (95% CI)				
*BCR::ABL1* ≤1%	37.93% (27.74%–48.13%)	41.54% (33.44%–49.65%)	42.29% (32.02%–52.43%)	41.11% (35.04%–47.17%)
MMR	12.64% (5.66%–19.63%)	24.84% (17.56%–31.74%)	25.30% (16.54%–34.57%)	13.33% (9.24%–17.64%)
12 months, % (95% CI)				
*BCR::ABL1* ≤1%	39.08% (28.83%–49.33%)	50.70% (42.48%–58.93%)	45.85% (35.27%–55.84%)	52.22% (46.02%–58.33%)
MMR	19.54% (11.21%–27.87%)	33.12% (25.36%–40.84%)	31.62% (21.55%–40.68%)	18.89% (14.14%–23.80%)

CI, confidence interval; CP-CML, chronic-phase chronic myeloid leukemia; IS, international scale; MMR, major molecular response.

#### Patients with CP-CML without baseline response

After MAIC adjustment, the combined sample size of PACE and OPTIC decreased from 343 to an ESS of 304 among those without a baseline response. Once patient characteristics were adjusted in the pooled PACE and OPTIC trials to match trials of asciminib, the response rate was calculated using the MAIC weight for each patient and the difference in rate was calculated using a Z-test for two independent proportions. Patients without a baseline response had higher response rates with ponatinib than with asciminib for both efficacy endpoints of *BCR::ABL1* ^IS^ ≤1% and MMR (**Table 4**) by both 6 and 12 months. By 12 months, the rate difference between asciminib and MAIC-adjusted ponatinib was 9.33% (95% CI: 0.79%–17.86%) for *BCR::ABL1* ^IS^ ≤1% in favor of ponatinib and 6.84% (95% CI: –0.95%–14.62%) for MMR in favor of ponatinib.

**Table 4:** Comparison of *BCR::ABL1* ^IS^ ≤1% and MMR by 6 and 12 months among patients with CP-CML without baseline response following MAIC adjustment.

Intervention	ASCEMBL + phase 1	PACE + OPTIC unadjusted	PACE + OPTIC MAIC-adjusted	Rate difference MAIC-adjusted
	Asciminib	Ponatinib	Ponatinib	Ponatinib vs. asciminib
Sample size	N=229	N=343	ESS=304.97	
6 months, % (95% CI)				
*BCR::ABL1* ^IS^ ≤1%	40.17% (33.82%–46.52%)	41.98% (36.76%–47.21%)	49.90% (44.29%–55.51%)	9.73% (1.25%–18.20%)
MMR	20.49% (15.43%–25.56%)	22.16% (17.76%–26.55%)	28.12% (23.07%–33.16%)	7.62% (0.48%–14.77%)
12 months, % (95% CI)				
*BCR::ABL1* ^IS^ ≤1%	46.29% (39.83%–52.75%)	47.52% (42.24%–52.81%)	55.61% (50.04%–61.19%)	9.33% (0.79%–17.86%)
MMR	28.28% (22.63%–33.93%)	28.28% (23.51%–33.05%)	35.11% (29.76%–40.47%)	6.84% (–0.95%–14.62%)

CI, confidence interval; CP-CML, chronic-phase chronic myeloid leukemia; ESS, effective sample size; IS, international scale; MAIC, matching-adjusted indirect comparison; MMR, major molecular response.

The model included the majority of the patients with CP-CML with or without the T315I mutation in the 4 selected trials. With the exception of MMR by 12 months, the superior performance in *BCR::ABL1* ^IS^ ≤1% and MMR for ponatinib was statistically significant compared with asciminib by both 6 and 12 months.

#### Patients with CP-CML with T315I mutation subgroups

Results by T315I mutation status were only available in the phase 1 study for asciminib, as patients with T315I mutations were ineligible for the ASCEMBL trial. Thirteen of 28 patients with CP-CML and the T315I mutation were ponatinib naive. In patients with the T315I mutation, response rates with ponatinib were consistently and significantly higher than with asciminib for both efficacy endpoints of *BCR::ABL1* ^IS^ ≤1% and MMR (**Table 5**) by both 6 and 12 months. By 12 months, ponatinib was 43.54% (95% CI: 22.20%–64.87%) and 47.37% (95% CI: 28.72%–66.02%) higher than asciminib for *BCR::ABL1* ^IS^ ≤1% and for MMR, respectively, after MAIC. Among the patients with the T315I mutation, the proportion of patients with *BCR::ABL1* ^IS^ ≤1% was over 2.5 times greater in the ponatinib group compared with the asciminib group by 12 months. In addition, the proportion of patients achieving MMR was over 4.5 times greater in the ponatinib group compared with the asciminib group by 12 months.

**Table 5:** Comparison of *BCR::ABL1* ^IS^ ≤1% and MMR by 6 and 12 months among patients with CP-CML with T315I mutation following MAIC.

Intervention	Phase 1	PACE + OPTIC pre-MAIC	PACE + OPTIC MAIC-adjusted	Rate difference MAIC-adjusted
	Asciminib	Ponatinib	Ponatinib	Ponatinib vs. asciminib
Sample size	N=24	N=81	ESS=53.43	
6 months, % (95% CI)				
*BCR::ABL1* ^IS^ ≤1%	25.00% (7.68%–42.32%)	58.02% (47.28%–68.77%)	66.26% (53.58%–78.94%)	41.26% (19.79%–62.73%)
MMR	12.50% (0.00%–25.73%)	37.04% (26.52%–47.55%)	46.21% (32.84%–59.58%)	33.71% (14.90%–52.52%)
12 months, % (95% CI)				
*BCR::ABL1* ^IS^ ≤1%	25.00% (7.68%–42.32%)	64.20% (53.76%–74.64%)	68.54% (56.08%–80.99%)	43.54% (22.20%–64.87%)
MMR	12.50% (0.00%–25.73%)	49.38% (38.49%–60.27%)	59.87% (46.72%–73.01%)	47.37% (28.72%–66.02%)

CI, confidence interval; CP-CML, chronic-phase chronic myeloid leukemia; ESS, effective sample size; IS, international scale; MAIC, matching-adjusted indirect comparison; MMR, major molecular response.

#### Patients with CP-CML without the T315I mutation

In patients without the T315I mutation, after MAIC, the proportions of patients with MAIC-adjusted *BCR::ABL1* ^IS^ ≤1% were higher for ponatinib vs. asciminib by the 6-month (4.95% [95% CI: −4.50%–14.41%]) and 12-month (4.77% [95% CI: –4.74%–14.29%]) timepoints (**Table 6**). The proportion of patients achieving MAIC-adjusted MMR was not different for ponatinib vs. asciminib by 12 months (−1.49% [95% CI: −10.01%–7.02%]) or by 6 months (2.48% [95% CI: −5.35%–10.31%]). Overall, the *BCR::ABL1* ^IS^ ≤1% and MMR response was slightly more favorable for ponatinib treatment in patients without the T315I mutation but not statistically significant.

**Table 6:** Comparison of *BCR::ABL1* ^IS^ ≤1% and MMR by 6 and 12 months among patients with CP-CML without T315I mutation following MAIC.

Intervention	ASCEMBL + phase 1	PACE + OPTIC pre-MAIC	PACE + OPTIC MAIC-adjusted	Rate difference MAIC-adjusted
	Asciminib	Ponatinib	Ponatinib	Ponatinib vs. Asciminib
Sample size	N=205	N=262	ESS=218.65	
6 months, % (95% CI)				
*BCR::ABL1* ^IS^ ≤1%	41.95% (35.20%–48.71%)	37.02% (31.18%–42.87%)	46.90% (40.29%–53.52%)	4.95% (–4.50%–14.41%)
MMR	21.36% (15.95%–26.78%)	17.56% (12.95%–22.16%)	23.84% (18.20%–29.49%)	2.48% (–5.35%–10.31%)
12 months, % (95% CI)				
*BCR::ABL1* ^IS^ ≤1%	48.78% (41.94%–55.62%)	42.37% (36.38%–48.35%)	53.55% (46.94%–60.16%)	4.77% (–4.74%–14.29%)
MMR	30.00% (23.94%–36.06%)	21.76% (16.76%–26.75%)	28.51% (22.52%–34.49%)	–1.49% (–10.01%–7.02%)

CI, confidence interval; CP-CML, chronic-phase chronic myeloid leukemia; ESS, effective sample size; IS, international scale; MAIC, matching-adjusted indirect comparison; MMR, major molecular response.

#### Sensitivity analysis: patients with non-T315I mutation with baseline *BCR::ABL1* ^IS^ >10%

Comparisons of *BCR::ABL1* ^IS^ ≤1% and MMR among patients with CP-CML without the T315I mutation with baseline *BCR::ABL1* ^IS^ >10% are presented in **Supplementary Table S2**. For asciminib, only the phase 1 asciminib trial could be included in the sensitivity analysis, as the ASCEMBL trial did not report cumulative *BCR::ABL1* ^IS^ ≤1% or MMR for this subgroup. After MAIC adjustment, the combined sample size of PACE and OPTIC decreased from 209 to an ESS of 204. After MAIC adjustment on age, sex, race, ECOG performance score and number of prior TKIs, the proportion of patients reaching *BCR::ABL1* ≤1% by 12 months was 18.8% greater in the ponatinib trials (33.94% [95% CI: 27.45%–40.42%]) compared with the phase 1 asciminib trial (28.57% [95% CI: 14.91%–42.23%]), with a rate difference of 5.37% (95% CI: –9.76%–20.49%), which was not statistically significant. The proportion of patients reaching MMR by 12 months was 22.7% greater in the ponatinib trials (17.53% [95% CI: 12.32%–22.73%]) compared with the phase 1 asciminib trial (14.29% [95% CI: 3.70%–24.87%]), with a rate difference of 3.24% (95% CI: –8.56%–15.03%), which was not statistically significant.

## Discussion

Based on the four identified clinical trials for ponatinib and asciminib, patients receiving ponatinib had higher response rates than those receiving asciminib after adjusting for prognostic factors and effect modifiers by MAIC in both outcomes (*BCR::ABL1* ^IS^ ≤1% and MMR) for most of the 6- and 12-month timepoints. In the MAIC analysis of patients with CP-CML without baseline response, ponatinib showed a nonsignificantly higher MMR and significantly higher *BCR::ABL1* ^IS^ ≤1% by 12 months compared with asciminib. Both outcomes were significantly higher in ponatinib by 6 months.

Among the T315I subgroup, ponatinib response rates were significantly higher than those with asciminib. Patients treated with ponatinib showed greater *BCR::ABL1* ^IS^ ≤1% and MMR response by 6 months and significantly greater responses by 12 months. These data suggest that ponatinib is effective in treating patients with the T315I mutation. Asciminib was approved more recently than ponatinib; therefore, some patients in the asciminib trial were pretreated with ponatinib whereas none of the ponatinib-treated patients were pretreated with asciminib. In the phase I trial, 54% and 30% of the patients with and without the T315I mutation, respectively, had previously received ponatinib (18). Because the individual patient-level data for the asciminib trials were not available for this study, we were not able to extract data by *BCR::ABL1* transcript level and by ponatinib pretreatment from trials of asciminib simultaneously for the MAIC analysis. Interestingly, the positive antineoplastic synergistic effects of asciminib and ponatinib in patients with T315I-mutated CP-CML have been demonstrated in CML cell lines (25); however, the potential influence of ponatinib pretreatment is unclear. In a 12-month follow-up of the phase I asciminib trial, four of the 17 (24%) evaluable patients with T315I mutations regardless of prior ponatinib exposure achieved MMR, while only one of six (17%) patients with the T315I mutation who were resistant or intolerant to ponatinib achieved MMR (18). Based on this report, the efficacy of asciminib in patients resistant or intolerant to ponatinib was found to be slightly inferior to that in the overall patients with the T315I mutation regardless of ponatinib exposure, although the sample sizes of these subgroups were quite small (18). Overall, the asciminib trials had a relatively lower sample size (n=24) in the T315I subgroup compared with the ponatinib trials (n=81).

Among patients who develop resistance to frontline or second-generation TKIs, changing to another second- or later-generation TKI is the necessary course. Resistance is often accompanied by mutations in the BCR::ABL protein, where sensitivities to different TKIs vary; therefore, the heterogeneity of co-mutations in BCR::ABL1 may impact the outcomes of treatment. In our study, it is unclear based on existing data from available trials how specific mutations impact the efficacy of ponatinib and asciminib.

Among patients without T315I mutation, the magnitude of the favorable effect of ponatinib was reduced compared with the overall patient population with or without T315I mutation. However, the results still favored ponatinib in most of the outcomes compared with asciminib. Among patients without T315I mutation and with an unfavorable baseline *BCR::ABL1* ^IS^ level (*BCR::ABL1* ^IS^ >10%), response rates were higher with ponatinib in the subgroup of patients with non-T315I mutation alone, although not statistically significant. The superior performance of ponatinib compared with asciminib in patients with T315I mutation in our MAIC is predicted and has been observed in cross-trial comparisons (26). Ponatinib is structurally designed with a carbon-carbon triple bond affording inhibition of native BCR::ABL1 as well as single-point mutants thereof, including the ABL1-T315I kinase domain gatekeeper mutation (27, 28). In the United States, asciminib is approved for use in patients with T315I-mutated CP-CML; however, the recommended dose is considerably higher (26).

In a SLR and comparative analysis, third-line ponatinib treatment was compared against 2G TKIs using study-level data (29). From this study, ponatinib has shown superior efficacy to all of the other 2G TKIs in patients with CP-CML. With the approval of asciminib in 2021, a comparison between ponatinib and asciminib was conducted with study-level data without adjustment (30). The comparison showed slightly higher efficacy of ponatinib compared with asciminib in the overall population of patients without baseline response and significantly higher efficacy of ponatinib treatment compared with asciminib in patients with the T315I mutation without adjusting for baseline variables. For this study, we used patient-level data from the PACE and OPTIC ponatinib trials, which allowed us to conduct the population-matched adjusted analyses to balance the baseline differences between different ponatinib and asciminib trials. Our results showed superior response rates with ponatinib in the overall patient population and in patients with the T315I mutation without a baseline response.

With similar approaches, a previous MAIC study compared asciminib against 2G TKIs (nilotinib and dasatinib) and ponatinib using patient-level data from the ASCEMBL trial and compared the adjusted results with the published data for ponatinib (24). Unlike our analyses, which included both the PACE and OPTIC trials for ponatinib and both the phase 1 and ASCEMBL trials for asciminib, the previous study only included the PACE trial for ponatinib and the ASCEMBL trial for asciminib for the MAIC analysis (31). Due to the heterogeneity between trials of ponatinib and asciminib, the ESS dropped drastically from 157 to 31 patients with asciminib treatment (n=270 for ponatinib) in the previous study, which is considerably lower than the ESS of our main analysis (ESS=304 for ponatinib; n=229 for asciminib). In addition, the study did not include patients with the T315I mutation. Contrary to our results, the previous study with a much lower ESS showed statistically significant improvements in MMR at both 6 months (relative risk [RR] = 1.55, 95% CI: 1.02–2.36) and 12 months (RR = 1.48, 95% CI: 1.03–2.14) with asciminib compared with ponatinib (31).

For treatment options, efficacy, safety, patient adherence, and economic burdens should be considered to ensure a patient-centric strategy. In the PACE study, the exposure-adjusted incidences of new arterial occlusive event (AOE) rates were 15.8 and 4.9 per 100 patient-years in years 1 and 5, respectively (17). In the OPTIC trial, response-based dosing strategies were explored to maximize response while minimizing toxicity. Patients in the 45- and 30-mg dose cohorts were required, once efficacy thresholds were reached (*BCR::ABL1* ^IS^ ≤1%), to have their dose reduced to 15 mg, with no dose reductions in the 15-mg cohort based on efficacy thresholds. Independently confirmed grade ≥3 treatment-emergent AOEs were found in five of 94 patients with a 45-mg initiation dose of ponatinib (16). Despite these protocol-mandated dose reductions, efficacy with ponatinib was sustained in the OPTIC trial. The response-based dose strategy demonstrated an improved benefit-to-risk in the third- and fourth-line settings and identifies the 45-mg starting dose cohort as having the optimal benefit-to-risk profile (16, 32).

In the phase 1 asciminib trial, AOEs were found in 10 of 115 patients, with five grade ≥3 (33). Contrary to the consistent single-dose or response-based dose reduction treatment for patients both with or without the T315I mutation, the indicated dose of asciminib for patients with the T315I mutation is five times higher than the dose for patients without the T315I mutation (400 mg/day vs 80 mg/day) in the US prescribing information. In the phase 3 ASCEMBL trial, 3.2% of patients in the asciminib arm had AOEs (20). Although asciminib showed a better overall safety profile than the trial comparator, bosutinib, the toxicity with the higher asciminib dose in patients with the T315I mutation may need to be considered and evaluated when making treatment decisions (18, 20). Patient adherence and economic burden should also be considered when prescribing higher doses of asciminib in patients with the T315I mutation. Further in-depth studies are needed to compare patient adherence, patient-reported outcomes, and health-care utilization between ponatinib and asciminib in real-world practice.

In our MAIC analysis, ponatinib was found to have higher response rates than asciminib. For both *BCR::ABL1* ^IS^ ≤1% and MMR outcomes up to a year, ponatinib showed significant favorable response among patients with CP-CML without baseline response. Patients with T315I mutations were found to have even higher response rates to ponatinib treatment; therefore, greater favorable differentiation was observed in patients with ponatinib treatment compared with those with asciminib treatment. Further long-term outcomes, such as survival outcomes, could be compared and add value to the benefit-to-risk profile when more mature data are available from the ASCEMBL trial.

Third-generation TKIs are the preferred treatment option in patients with CML with resistance to second-generation TKIs or with the T315I mutation. The efficacy and safety of ponatinib have been established in phase 2 trials (16, 17) and years of clinical experience. Asciminib received regulatory approval for the treatment of CML in 2021 and therefore has limited long-term data and experience, related only to the phase 1 study. Although there are no head-to-head trials, results from prospective trials indicate that patients with T315I-mutated CML treated with ponatinib have better outcomes than those treated with asciminib (16–18, 20, 34–36). Compared with second-generation TKIs, ponatinib has shown better survival in third-line CML therapy, a finding not observed with asciminib considering the shorter follow-up from clinical trials (26, 37). The results of this MAIC analysis support clinical findings and suggest that ponatinib may provide greater benefit in patients with the T315I mutation.

### Limitations

Resistance and intolerance are potentially critical for *BCR::ABL1* ^IS^ ≤1% and MMR. Unfortunately, we were not able to include these variables in the MAIC, as the ponatinib trials had a greater proportion of patients with resistant disease and did not have a number of patients with intolerance to match those in the asciminib trials. The data from ASCEMBL and the phase 1 asciminib trial were based on the aggregated data in the public domain. Therefore, the comparison between ponatinib and asciminib is limited by the availability of the published data. In addition, the MAIC was limited by the baseline measures that were available for all included studies. Therefore, baseline characteristics such as duration of CP-CML, sequence of prior TKIs, and detailed history of resistance or intolerance to a specific TKI could not be included to account for differences in enrolled populations. Some of the subgroup analyses contained low sample sizes due to data availability. The results from these models should be interpreted with caution. Likewise, in the subgroup analysis of patients with baseline response >10%, a small ESS may decrease reliability and increase the confidence interval, resulting in a low likelihood of detecting treatment differences. Finally, the efficacy outcomes selected in this study are the key primary or secondary endpoints of the trials and are the only ones that were consistently reported across the trials. Safety outcomes were not included in the study due to inconsistent reporting methods and differential dosing or dose reduction in some of the trials. However, the different safety profiles should be considered according to a patient-centered approach.

## Conclusions

Using a MAIC approach to analyze data from key clinical trials for ponatinib and asciminib, with adjustment and balancing of patient characteristics, response rates as measured by *BCR::ABL1* ^IS^ ≤1% and MMR were higher in patients with or without a T315I mutation and without baseline response when treated with ponatinib compared with asciminib by both 6 months and 12 months posttreatment. In patients with T315I mutation, ponatinib consistently showed significantly favorable MMR compared with asciminib.

## Summary

This MAIC analysis of clinical trial data evaluated ponatinib and asciminib in patients with R/R CP-CML who failed ≥1 second-generation TKI or had the T315I mutation. After adjusting for baseline characteristics, ponatinib showed consistently higher *BCR::ABL1* ^IS^ ≤1% and MMR rates than asciminib by 6 and 12 months. In overall patients without a baseline response and regardless of their T315I mutation status, *BCR::ABL1* ^IS^ ≤1% and MMR rates were statistically higher in most of the comparisons with ponatinib by 12 months. In patients with the T315I mutation, significant favorable efficacy outcomes were observed with ponatinib versus asciminib.

## Data Availability

The data sets, including the redacted study protocol, redacted statistical analysis plan, and individual participant data of the completed study supporting the results reported in this article, will be made available within three months from initial request to researchers who provide a methodologically sound proposal. The data will be provided after de-identification, in compliance with applicable privacy laws, data protection, and requirements for consent and anonymization.

## Footnotes

1 Referenced with permission from the NCCN Clinical Practice Guidelines in Oncology (NCCN Guidelines®) for Chronic Myeloid Leukemia V.1.2025. © National Comprehensive Cancer Network, Inc. 2024. All rights reserved. Accessed August 8, 2024. To view the most recent and complete version of the guideline, go online to NCCN.org. NCCN makes no warranties of any kind whatsoever regarding their content, use or application and disclaims any responsibility for their application or use in any way.
