# Supplementary material for: Ponatinib vs. asciminib in post–second-generation tyrosine kinase inhibitor therapy for chronic-phase chronic myeloid leukemia: a matching-adjusted indirect comparison
Source: Front Oncol. 2024 Nov 20;14:1455378. doi: 10.3389/fonc.2024.1455378 (PMC11615674; doi:10.3389/fonc.2024.1455378)
Supplement: Supplementary file 1 [file Table1.docx]

Supplementary Material

# Supplementary Tables

## Table S1. Comparison of patient characteristics before and after MAIC (phase 1 asciminib vs. PACE and OPTIC) in a subgroup of patients with T315I mutation

|  | **Phase 1**  **asciminib^a^** | **PACE + OPTIC**  **ponatinib**  **unadjusted** | **PACE + OPTIC**  **ponatinib**  **MAIC-adjusted^b^** |
| --- | --- | --- | --- |
| Sample size | 141 | 88 | ESS: 53.43  PACE: 43.52  OPTIC: 9.91 |
| Mean age, years (SD) | 55.5^c^ | 50.2 | 55.5 |
| Sex, male | 54.5% | 73.9% | 54.5% |
| Race, White | UNK | 69.3% | Unadjusted |
| ECOG performance status, 1 or 2 | 27.3% | 26.1% | 27.3% |
| Mean prior TKIs | 2.7^d^ | 2.3 | 2.7 |
| *BCR::ABL1*^IS^ level >10% | 43.3% | 75.0% | 43.3% |

Abbreviations: ECOG, Eastern Cooperative Oncology Group; ESS, effective sample size; IS, international scale; MAIC, matching-adjusted indirect comparison; SD, standard deviation; TKI, tyrosine kinase inhibitor; UNK, unknown

^a^The weighted results from phase 1 and ASCEMBL trials were used as the reference of the MAIC analysis

^b^MAIC analysis was conducted by using patient-level data from PACE and OPTIC trial which were matched against the combined results of phase 1 asciminib in all of the patient characteristics listed in the table. Exceptions were noted in the table

^c^Only median number was available in the phase 1 asciminib study

^d^Prior TKI number in phase 1 asciminib trial was estimated based on the categorical data

## Table S2. Comparison of *BCR::ABL1*^IS^ ≤1% and MMR by 6 and 12 months among patients with CP-CML without T315I mutation with baseline *BCR::ABL1*^IS^ >10%

|  | **Phase 1** | **PACE + OPTIC unadjusted** | **PACE+OPTIC**  **MAIC-adjusted** | **Rate difference**  **MAIC-adjusted** |
| --- | --- | --- | --- | --- |
| Intervention | Asciminib | Ponatinib | Ponatinib | Ponatinib vs. asciminib |
| Sample size | N=42 | N=209 | ESS=204 |  |
| **6 months, % (95% CI)** | | | | |
| *BCR::ABL1*^IS^ ≤1% | 23.81% (10.93%–36.69%) | 30.14% (23.92%–36.36%) | 28.91% (22.70%–35.12%) | 5.10% (–9.20%–19.40%) |
| MMR | 7.14% (0.00%–14.93%) | 13.40% (8.78%–18.02%) | 14.48% (9.66%–19.30%) | 7.34% (–1.82%–16.50%) |
| **12 months, % (95% CI)** | | | | |
| *BCR::ABL1*^IS^ ≤1% | 28.57% (14.91%–42.23%) | 35.41% (28.92%–41.89%) | 33.94% (27.45%–40.42%) | 5.37% (–9.76%–20.49%) |
| MMR | 14.29% (3.70%–24.87%) | 16.75% (11.68%–21.81%) | 17.53% (12.32%–22.73%) | 3.24% (–8.56%–15.03%) |

Abbreviations: CI, confidence interval; CP-CML, chronic-phase chronic myeloid leukemia; ESS, effective sample size; IS, international scale; MAIC, matching-adjusted indirect comparison; MMR, major molecular response

**
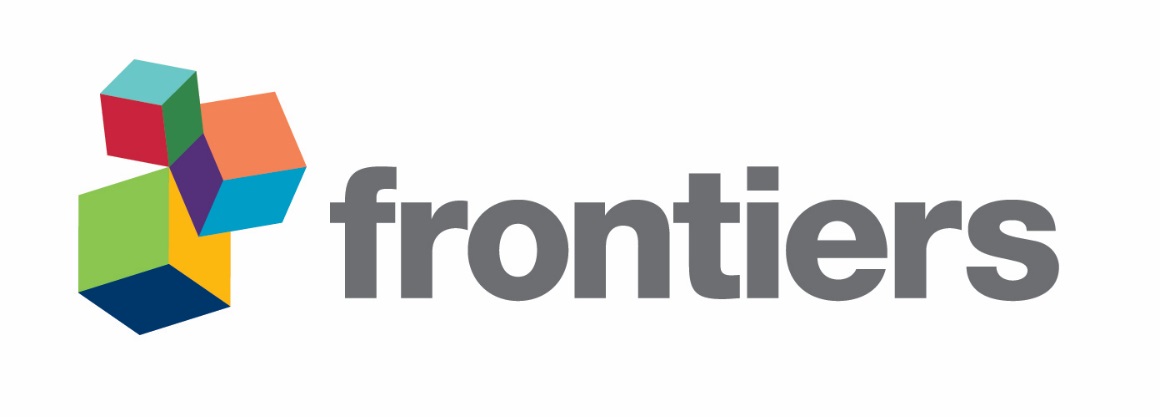
**
